# CONNECT for quality: protocol of a cluster randomized controlled trial to improve fall prevention in nursing homes

**DOI:** 10.1186/1748-5908-7-11

**Published:** 2012-02-29

**Authors:** Ruth A Anderson, Kirsten Corazzini, Kristie Porter, Kathryn Daily, Reuben R McDaniel, Cathleen Colón-Emeric

**Affiliations:** 1 School of Nursing, Duke University, Trent Drive, DUMC 3322, Durham, NC 27710, USA; 2 Department of Management Science & Information Systems, McCombs School Business, The University of Texas at Austin, 2100 Speedway, CBA 6.454, Austin, TX 78712, USA; 3 Division of Geriatrics, Department of Medicine, School of Medicine, Duke University Trent Drive, DUMC Box 3003, Durham, NC 27710, USA

## Abstract

**Background:**

Quality improvement (QI) programs focused on mastery of content by individual staff members are the current standard to improve resident outcomes in nursing homes. However, complexity science suggests that learning is a social process that occurs within the context of relationships and interactions among individuals. Thus, QI programs will not result in optimal changes in staff behavior unless the context for social learning is present. Accordingly, we developed CONNECT, an intervention to foster systematic use of management practices, which we propose will enhance effectiveness of a nursing home Falls QI program by strengthening the staff-to-staff interactions necessary for clinical problem-solving about complex problems such as falls. The study aims are to compare the impact of the CONNECT intervention, plus a falls reduction QI intervention (CONNECT + FALLS), to the falls reduction QI intervention alone (FALLS), on fall-related process measures, fall rates, and staff interaction measures.

**Methods/design:**

Sixteen nursing homes will be randomized to one of two study arms, CONNECT + FALLS or FALLS alone. Subjects (staff and residents) are clustered within nursing homes because the intervention addresses social processes and thus must be delivered within the social context, rather than to individuals. Nursing homes randomized to CONNECT + FALLS will receive three months of CONNECT first, followed by three months of FALLS. Nursing homes randomized to FALLS alone receive three months of FALLs QI and are offered CONNECT after data collection is completed. Complexity science measures, which reflect staff perceptions of communication, safety climate, and care quality, will be collected from staff at baseline, three months after, and six months after baseline to evaluate immediate and sustained impacts. FALLS measures including quality indicators (process measures) and fall rates will be collected for the six months prior to baseline and the six months after the end of the intervention. Analysis will use a three-level mixed model.

**Discussion:**

By focusing on improving local interactions, CONNECT is expected to maximize staff's ability to implement content learned in a falls QI program and integrate it into knowledge and action. Our previous pilot work shows that CONNECT is feasible, acceptable and appropriate.

**Trial Registration:**

ClinicalTrials.gov: NCT00636675

## Background

Efficacy trials [1] have shown that interventions that reduce multiple fall risk factors also lower fall rates, recurrent falls, and injurious falls in nursing home residents [2-5]. However, interventions in these trials were completed by specially-hired external study staff; prior attempts to move fall risk factor reduction into everyday practice by in-house staff have not been successful [6-10]. Quality Improvement (QI) interventions [11,12] are the current gold standard for introducing evidence-based care into nursing homes. These QI interventions provide the content for reducing falls but do not ensure that the processes needed to successfully implement fall reduction strategies are in place [13].

A particular barrier for QI programs is that they do not fully address staff interdependencies inherent in care for falls or other geriatric syndromes. These syndromes result from multiple risk factors and require multifactorial, interdisciplinary interventions to improve outcomes [14]. For example, falls efficacy trials have intervened on gait, incontinence, sensory impairment, cognitive impairment, psychoactive medications, orthostasis, toileting, and environmental factors [15]. Reducing multiple risk factors may be difficult because it requires many staff members to have strong connections that permit effective information flow and problem-solving from varied perspectives. Thus, an intervention is needed to help nursing home staff establish relationship networks and communication channels to support the new practices introduced by QI programs.

Complexity science provides useful insights for addressing barriers to effective staff interdependence. It suggests that management practices (MPs) that facilitate self-organization are most likely to enhance a nursing home's ability to achieve high-quality outcomes [16-19]. Through self-organization, staff interact and mutually adjust their behaviors using what they learn from each other to cope with changing care and environmental demands [17]. Relationship-oriented MPs, such as open communication, participation in decision-making, and teamwork, result in better resident outcomes, possibly through better staff connections and information flow [20]. Our recent case studies [19] identified additional MPs associated with enhanced staff connections, and these MPs are particularly suited to foster effective interdependence needed to care for people with geriatric syndromes such as falls. Staff at all levels used these MPs, but only erratically. Therefore, an intervention that fosters systematic use of these relationship-oriented MPs would facilitate more effective interdependence by creating networks and communication channels for learning together, exchanging care information, and solving problems. Based on complexity science [21,22] and our prior research [7,16,19,23-29], we developed the CONNECT intervention, which we propose will create the foundation (processes) for staff to effectively implement QI interventions (content) to reduce falls through more effective self-organization. Because of interdependence and self-organization, the intervention must be delivered within the social context in which individuals work, rather than to individuals alone. Thus, a cluster randomization is needed.

CONNECT is a multi-component intervention that helps staff: learn new strategies to improve day-to-day interactions; establish relationship networks for creative problem solving; and sustain newly acquired interaction behaviors through mentorship. Complexity science and empirical research suggest that interaction patterns determine information flow, knowledge transfer, and capacity to monitor behaviors in healthcare settings [16,18,30]. In a preliminary test of CONNECT, we found support for the hypothesis that the intervention would improve staff interactions and reduce falls [31]. We propose that CONNECT, when combined with a content focused falls QI program (FALLS), will result in better resident outcomes when compared to FALLS alone. We chose falls for this test of CONNECT because: falls rates are high in nursing homes [32,33]; accepted practice guidelines and fall prevention programs exist [34-37]; and there is ample evidence from efficacy trials that multi-factorial risk reduction interventions reduce fall rates [3-5,13,38].

The study aims are to:

1. Compare the impact of the CONNECT intervention plus a falls reduction QI intervention (CONNECT + FALLS) to the falls reduction QI intervention alone (FALLS) on fall-related process measures in nursing home residents.

2. Compare the impact of CONNECT + FALLS to FALLS alone on fall-rates in nursing home residents, and determine whether these are mediated by the change in fall-related process measures.

3. Compare the impact of CONNECT + FALLS to FALLS alone on complexity science measures as reported by nursing home staff and determine whether these mediate the impact on fall-related process measures and fall rates.

To complete these aims we will use a cluster randomized, controlled trial design with the facility-level change in fall and complexity science measures as the primary outcomes. Because the CONNECT and FALLS interventions both encourage facility-wide change in staff behavior, randomization will occur at the facility level and subjects (nursing home staff and residents) are clustered within homes.

### Significance

Improving resident outcomes in NHs remains a national priority. While effective practices are known from efficacy trials, there is a lack of knowledge about how NH staff can implement these practices [2-5,13]. Centers for Medicare and Medicaid Services contracts with QI organizations to implement QI programs, including QI collaboratives, educational programs, and toolkits to reduce geriatric syndromes such as falls, pressure ulcers, incontinence, pain, delirium, and depression [39-41]. Unfortunately, such efforts have not resulted in the expected improvements [6,7,9,42]. Complexity science suggests that a major barrier to the effectiveness of QI programs is their content focus; they do not impact the processes needed to actually implement practice change. The CONNECT for Quality study is significant because it will test a novel intervention that attempts to create the foundation needed for nursing home staff to implement content learned in QI programs such as FALLS. Thus, CONNECT has the potential to have a broad and far-reaching impact on QI efforts nationally and influence care for multiple geriatric syndromes.

Of further significance, this study uses existing staff to improve resident care, without requiring additional resources. CONNECT, which targets local interactions among staff, strengthens the interdependencies among staff and also addresses other common barriers to interdisciplinary problem solving, such as omitting Licensed Practical Nurses and Nurse Aids (NAs) from decision making [24,25,43], poor communication between provider groups [23], and overreliance on hierarchical management [24,30,44-46]. CONNECT, if successful, thus has the potential to be generalizable to real-world nursing home settings by enhancing existing staff capacity to learn and improve.

Further, this study is significant because it puts the tools of change into the hands of direct- care staff. CONNECT will establish networks for new information about fall risk factor reduction to spread throughout the nursing home. These networks are critical because NAs provide 80-90% of the hands-on care to residents [47], and they often are the first to observe early signs of fall risk [48]. Yet, NAs frequently lack the interactions with the multi-disciplinary team needed to intervene effectively [25,49]. CONNECT will create opportunities for more rapid information exchange and problem solving among multiple disciplines and will increase the likelihood that the NA will carry out appropriate fall prevention care.

In our prior work [16-19,23-25,27,28,30,50,51], we found that both managers and staff can use MPs to influence self-organization and produce better quality of care. In CONNECT, staff will learn to consider three system parameters [22] derived from the theory of complex adaptive systems [22] to guide their use of nursing MPs. Relationship-oriented MPs in CONNECT, which are collectively called local interaction strategies, influence the three system parameters--connection between staff members, information exchange, and cognitive diversity. When staff use the MPs, they create and recreate meaning of events, change beliefs, foster creativity, and promote reflection on their performance [21,22]. For example, when staff members interact, they develop networks [52]. These new networks of connections allow local changes in behavior to result in system-wide change. When staff members interact they exchange information, which generates new understanding and knowledge [53,54]. With this knowledge, staff members learn, change behaviors [54], and become capable of accomplishing something new. Finally, cognitive diversity, the use of multiple perspectives to make sense of information [50,54], arises from interaction among people. The more diverse the individuals (*e.g*., varying roles, education, social or cultural backgrounds, age cohorts [52], and external collaborations [53]) the richer the interpretation of data, the more appropriate the decision making, and the more effective the action planning [50,51].

**Figure 1:**
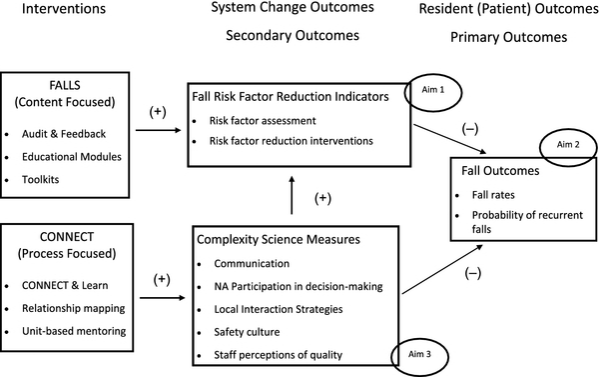
**Proposed Relationship between the FALLS and CONNECT Interventions**. The FALLS intervention is expected to provide nursing home staff members with the content needed to know what fall reduction assessments and interventions to use for residents at risk for falling. Increased use of these fall reduction assessments and interventions are expected, in turn, to reduce the fall rates and probability of recurrent falls among nursing home residents. The CONNECT intervention, on the other hand, is expected to directly reduce the fall rates as well as increase the staff's use of the fall reduction assessment and interventions, thus having a greater impact of fall reduction than the FALLS intervention alone.

We propose that systematic use of these local interaction strategies to create relationship networks and channels of communication for learning together, exchanging information, and solving problems, is a prerequisite to the ability to effectively implement a fall reduction program. Based on complexity science theory, if we achieve expected changes in staff interactions, we will observe changes in measures of communication, participation in decision-making, relational coordination, psychological safety, and safety culture (Figure 1). These measures in turn are expected to be related to more effective fall risk factor reduction measures.

CONNECT is expected to work in combination with QI programs because CONNECT creates processes for group learning and implementation of evidence-based content introduced by the QI program. FALLS will include content on evidence-based practices found to reduce falls in efficacy trials [2-4]. Modifiable fall risk factors, suggested by clinical practice guidelines and Agency for Healthcare Research and Quality's fall management program are: orthostatic hypotension [55,56]; sensory impairment [57,58]; footwear; gait and assistive devices [4-59]; toileting needs [60]; environmental problems [61]; fall-related medications [62,63]; and vitamin D. [64-67] CONNECT is an important companion for QI interventions such as FALLS because it creates relationship networks and communication channels for learning, information exchange, and problem solving.

## Methods and design

In this cluster randomized, blinded trial, 16 nursing homes will be randomized to one of two study arms either to receive CONNECT + FALLS or FALLS alone. Subjects are clustered within nursing homes because the intervention addresses social processes and thus must be delivered within the social context, rather than to individuals. Nursing homes randomized to CONNECT + FALLS will first complete the CONNECT protocol, after which they will receive the FALLS protocol. Nursing homes randomized to FALLS alone will be offered CONNECT following data collection. Complexity science measures, to be completed by nursing home staff, will be collected at baseline, and three and six months after baseline to evaluate the immediate and sustained impact on system parameters. FALLS measures, collected from medical record review, will be collected longitudinally for the six months prior to baseline and the six months after the end of the intervention to allow adequate fall events to accrue. A five-year timeline is planned to complete the study. This study was review and approved by the Duke University Medical Center Institutional Review Board.

### Sample and setting

#### Nursing home recruitment and randomization

A sample of nursing homes will be drawn from 69 facilities in North Carolina that participate in Medicare and Medicaid and are within a 100-mile radius of Duke University. Nursing Home Compare Data [68] show that facilities in the sampling pool are not substantially different from national averages. The North Carolina Quality Improvement Organization, the Carolinas Center for Medical Excellence (CCME), will recruit for our study. They successfully recruited 38 nursing homes in the Principal Investigators' (PI) previous QI study [69].

Eligible nursing homes will be contacted in random order by CCME until 16 agree to participate. Because participation is voluntary, there is unavoidable potential for participation bias. To assess for this, we will compare participating and refusing nursing homes using available data such as size, ownership, and nursing staffing. To avoid long delays between recruitment and participation, we will recruit in waves of six, six, and four. Because chain-affiliation relates to care quality [70], we will stratify our randomization based on chain or non-chain affiliation to ensure equal balance for potential confounders such as corporate policies. Once the first six nursing homes (clusters) are recruited, they will be placed in strata and stripped of all identifiers except a study number. An independent investigator blinded to nursing home name and characteristics will assign block size based on the strata size; if only two nursing homes are in a strata block size, two will be used, otherwise block size will be randomly assigned at two, four, or (if applicable) six. The independent investigator will then randomize within blocks into study arms using a random number generator. The randomization sequence and block size will be concealed until interventions are assigned.

#### Resident sample

Eligibility criteria include: > 65 years of age; sustained a fall as defined by Minimum Data Set (MDS) criteria in the study period; and remained in the facility for at least 30 days after the fall event. This sampling strategy will allow us to measure fall risk factor reduction activities completed by the nursing home staff for their highest risk residents (i.e., known fallers). Previous studies suggest a fall rate of 1.5 falls/bed-year, of which 40% are recurrent fallers [32-59]. Of the approximately 1,600 residents in the study nursing homes, we estimate a resident pool of n = 1,440 unique fallers which exceeds our needed resident sample size of 800. Lists of residents who have fallen during the study period will be generated from the nursing home MDS and incident reports. A random sample of 50 unique residents from each nursing home will be selected for chart abstraction using a random number generator. Because this is a minimal risk study in which residents are not followed prospectively, we have obtained a waiver of informed consent.

#### Staff sample

Staff members who work with residents in a clinical capacity (e.g., registered nurses, licensed practice nurses, NAs, social workers, dietary, activities, physical and occupational therapists) on skilled and assisted living units will be eligible to participate. The only exclusion criterion is inability to understand English. Using current staff lists provided by the administrator, we will invite staff to participate. In our pilot studies, 80% to 84% of staff invited, participated in survey completion. Thus, we conservatively estimate that of about 960 staff members, 60% will participate in training and complete surveys for an estimated enrollment of 576 staff members. New employees will be invited to participate in CONNECT up to the fourth week of the intervention. Those joining later will be invited to enroll only to complete the cross-sectional staff interaction measures. Based on data from the 2000 U.S. Census, we project that staff racial composition will be 57% white, 39% African American, and 3% other races [71]. Our pilot studies included 34% underrepresented minority participants.

### Risks and challenges

A major challenge for nursing home research is the potential for staff turnover. Using a successful strategy from our prior studies, we will secure a written commitment from the nursing home administrator, director of nursing, and if relevant, a corporate representative, that the study will continue even if one or more top administrators leave. We also have designed this study to be robust to staff turnover by incorporating the CONNECT in-class learning sessions into the nursing home's orientation for new staff. Exploratory analyses will determine whether staff turnover affects the fall-related processes or fall rate measures. Another challenge for nursing home research is designing approaches that are appropriate and acceptable for all levels of staff, regardless of education and socio-economic background. We use storytelling, which is an efficient yet high-impact method of conveying information, infused with relevant nursing home cultural norms, values, and beliefs [72]. Because storytelling and role play are based on descriptive verse, they may attenuate learning barriers associated with low literacy and English as a second language [73].

### The interventions

CONNECT will be implemented over 12 weeks, followed by FALLS for an additional 12 weeks. The FALLS intervention is a modification of interventions previously tested by the PI [7,69] and is based on the Falls Management Program developed by the Agency for Healthcare Research and Quality [59,74]. Details of the intervention components, rationale, participants, and time required for CONNECT and FALLS are in Tables 1[75] and 2[75] respectively. All aspects of the interventions are applied to cluster level; even aspects that are delivered to individuals, address cluster level interactions. The complete text of the CONNECT and FALLS protocols are available on request to the authors.

**Table 1:** CONNECT protocol activities, rationale, who is involved and time required

CONNECT Protocols	Rationale/Outcome	Who	Time
**Learning Protocols**			

**CONNECT & Learn Protocols** CONNECT Basics (Session 1). Introduces local interaction strategies using storytelling and practice using role-play in context of falls prevention. CONNECT Advanced (Session 2). Brief review followed by focus on the more advanced strategies of cognitive diversity, using storytelling, role-playing, and discussion of participants' experiences in applying concepts.	Interdisciplinary learning facilitates skill acquisition, creation of new horizontal and vertical connections among staff, and learning through cognitive diversity.	RNs, LPNs, NAs, social work, activities, rehab, MD, NP; dietary, administration	2, 30 min sessions occurring 2 weeks apart (1.0 hrs total)

**In-House Facilitator Training Protocols** In-House Facilitator Class Training. In-house facilitators learn to facilitate interdisciplinary in-class learning and/or practice mentoring and problem-solving at the point of care to improve local interactions. Chance Encounter Mentoring Training. Researcher shadows the In-house facilitator trainee during the work day to identify mentoring opportunities and model 'chance encounter mentoring;' observe and advise trainee as s(he) practices the behaviors; and jointly problem solve (1 session of 1 hr). Support by research facilitators. The researcher contacts the in-house facilitators weekly for support and advising; in-house facilitators also have a phone number to call to seek help from research staff as needed.	Prepares in-house care and supervisory staff to build trust and maintain consistency of CONNECT with the local culture. Facilitates information exchange between nursing home staff and research staff. In-house facilitators develop self-efficacy in using chance encounters to model local interactions and to mentor staff.	Care staff or managers in clinical departments (e.g., nursing, social work, activities). Individuals self-selected with encouragement of study staff.	1, 1 hr learning session; Up to 1 hr of shadowing during regular work activities; 5, 10 min discussions (up to 2 hrs, 50 min total)

**Relationship Map Protocols**			

**Group-to-group maps** Session 1. Researcher assists staff to describe actual interactions between work groups (e.g., NAs, LPNs, SW, Dietary, etc.). Session 2. Researcher assists staff to depict new interaction patterns and develop guidelines for improved group-to-group interaction patterns.	Assists staff to make interaction patterns explicit (develop a group-to-group relationship map), and agree on guidelines for improved interactions.	Mid-level managers and selected LPNs, NAs.	1, 1-hr class; 1, 70 min class; 1 week apart (2 hrs, 10 min total)

**Individual-to-individual maps** Researcher assists staff to draw an individual 'relationship map' that defines his/her ideal interactions with selected co-workers; reviews strategies for improving interactions. Participants learn to self-monitor and record interactions using relationship maps (available on a laminated card) and paper/pencil recording sheets.	Assists staff to evaluate relationships. Self-monitoring reinforces and sustains newly acquired behaviors and provides a measure of adherence and behavior change.	All CONNECT participants	1, 30 min session (30 min total)

**Unit Based Mentoring Protocols**			

**Structured Mentoring (by Research Facilitator)** During the 2 weeks following each in-class session, the researcher engages each participant in a 10-min session to discuss and reflect on his/her experiences applying CONNECT concepts. The researcher uses a semi-structured guide to elicit concerns about using the strategies.	Facilitates authentic learning, which occurs only when learners can directly and independently apply concepts [75].	All CONNECT participants	2, 10 min sessions (20 min total)

**Chance Encounter Mentoring (by In-house Facilitator)** In-house facilitators engage in point-of-care discussions with staff to practice CONNECT behaviors and jointly problem solve, using the 'chance encounter' protocol. They record the number and descriptions of chance encounter mentoring sessions. At least 5 such encounters should occur daily during naturally occurring usual work activities.	Identifies staff concerns and barriers, facilitates ongoing learning about interaction, and strengthens sustainability of new behaviors. Facilitators learn to use existing time differently.	In-house Facilitators engage with floor staff in their department or work unit	1.25 hrs/day for in-house facilitator (37 hrs total)

**Table 2:** FALLS protocol activities, rationale, who is involved and time required

FALLS Protocols	Rationale/Outcome	Who	Time
FALLS Coordinator and Team Role			

Training Session Researcher reviews: 1) role of FALLS Coordinator and Team members; 2) Falls Management Program rationale and main components; 3) annotated slide presentation on practical aspects of fall prevention; 4) toolkit materials; 5) study expectations.	Falls Team members champion fall prevention, identify area to improve, monitor changes.	FALLS Coordinator, Falls Team, DON	1, 4 hrs session

Weekly FALLS Team teleconference Researcher contacts FALLS team weekly during 3-month intervention for problem-solving/discussion, and highlights a topic from the Fall Management Program in more depth. Topics include 1) staff fall prevention education; 2) medications and falls 3) patient and family fall education; 4) orthostatic hypotension; 5) vision assessment and intervention; 6) gait and balance assessment and intervention 7) environmental assessment and intervention; 8)challenging behavior management; 9) establishing a culture of safety; 10) audit and feedback; and 11) Wrap-up and re-setting goals	Reinforces key concepts of multi-factorial risk reduction, supports FALLS Coordinator and maintains enthusiasm.	FALLS Coordinator, and any other team members s/he wishes	11, 30 min sessions weekly (5.5 hrs total)

Staff Education			

Case-Based Modules (online and paper form) Nurse module. Covers impact, fall risk factor assessment and intervention focusing on orthostatics, gait, toileting, medications, environmental hazards. NA module. Covers fall risk factor identification and intervention focusing on gait, footwear, toileting, hip protectors, and environmental hazards. Prescriber/pharmacist module. Covers epidemiology/impact, risk factor assessment, risk factor reduction focusing on psychotropic medication reduction and Vitamin D.	Uses case-based learning to impart knowledge and change attitudes about multi-factorial fall risk reduction.	RNs, LPNs, NAs, MDs, NPs, PAs, Consultant Pharmacists and others (PT, SW, Activities etc)	30-60 min

Post-Fall Problem-solving			

Academic Detailing Nursing home frontline staff is invited to participate in consultations with the researcher and FALLS Coordinator regarding their most challenging residents with falls, modeling risk factor assessment and multi-factorial interventions. Sessions occur at each nursing station during the day and evening shifts.	Reinforces key concepts and promotes behavior change and interdisciplinary discussions [75].	Nurses, NAs, other interested staff	2, 20 min sessions (40 min total)

Audit and Feedback			

Feedback Report Report uses visual (bar graph) and written depictions of the nursing home's current practice on fall-related process and outcome measures, and how this compares with peer nursing homes. Researcher presents and explains the feedback report to FALLS Team.	Identifies areas for improvement, promotes behavior change [75].	FALLS team, others as desired by Falls Coordinator	30 min

Toolbox			

Morse Fall Scale: Validated scale that quantifies fall risk in nursing home residents; Nurse Fall Risk Reduction Worksheet: Prompts nurse to identify and modify reversible fall risk factors. Can be used for chart documentation; Prescriber/Pharmacist Medication Reduction Worksheet: Prompts consideration of dose reduction or discontinuation of high fall-risk medications, including lower risk substitution options; Environmental Checklist: Facilitates identification of hazards in resident room, bathroom, and common areas; Wheelchair maintenance log and stickers: Facilitates regular assessment and repair of wheelchair brakes; Fall Risk Fax Communication Form: Allows nurse/pharmacist to communicate concerns about medications with prescribers; Patient and Family Brochure: Describes interventions that the nursing home is using to reduce falls; Physician/Prescriber Brochure: Describes the fall reduction program and encourages review of medication reduction worksheets and faxes.	Provides modifiable tools to assist with communication, implementation, and documentation of multi-factorial risk reduction.	FALLS Coordinator determines dissemination	Voluntary

### Treatment fidelity

Our treatment fidelity protocols use the National Institutes of Health Behavior Change Consortium's [76] model of treatment fidelity.

### Design

To ensure design fidelity, we standardized the CONNECT and FALLS protocols to a specified dose in terms of number, frequency, and length of contact.

### Training

CONNECT and FALLS will be delivered by different research interventionists trained separately to minimize contamination. The protocol specifies training content, structured practice, and role-play exercises to ensure that interventionists' skills meet established standards.

### Delivery

To ensure that CONNECT and FALLS are delivered as intended, a research team member will observe the interventionists on a random schedule, completing standardized checklists. The interventionists and PIs will discuss the results and problem-solve barriers to adherence and repeat concepts and role-play as needed. We will track participants that complete study components. For CONNECT, we will use: contact summary sheets for each visit to a research site; databases for interventionists to record contacts with participants; and sign-in sheets to document participation in sessions. For FALLS, we will use: contact sheets to record each contact between interventionists and the Fall Team; sign-in sheets to document participation in post-fall problem-solving sessions; and databases to track completion of educational modules via requests for continuing education credit or certificate of completion.

### Receipt of treatment

For CONNECT, participants' self-monitoring of local interactions will provide a measure of adherence and behavior change. The class sessions will include discussion and practice during which skills can be systematically assessed. For FALLS, participants will complete post-tests in the educational modules.

### Enactment of skills

Researchers will systematically assess enactment when they shadow the in-house facilitators to observe how they practice mentoring behaviors. The researchers will also assess and record enactment by participants during structured mentoring. Finally, they will assess enactment by observing at least two orientation sessions in which the in-house facilitator delivers the in-class session to new employees. Fall risk reduction indictors will be used to measure enactment of the FALLS intervention.

### Recruitment and data collection procedures

#### Staff recruitment and consent

When we recruit nursing homes, administrators and directors of nursing will agree to include CONNECT & Learn sessions and/or FALLS modules as regular in-service training. In meetings (e.g., nurses meetings, CNA meetings), researchers will explain the study and invite staff to participate in the other aspects of the study (completing surveys, structured mentoring). Staff not attending meetings will be approached individually. A research team member will answer questions and obtain written informed consent.

#### Staff incentives

As in our prior studies, we will offer an exit-interview consultation [77] during which we will share study results with participants. Continuing education credits or a certificate of completion will be given to staff for completing CONNECT & Learn sessions and/or FALLS educational modules. Everyone completing both learning sessions and staff surveys will receive practical items (water bottles, tote bags) with the study logo.

#### Data collection from staff

Data will be collected from enrolled staff at baseline, and at three and six months following baseline. Because some nursing home staff may have low literacy or English as a second language, obtaining reliable data will require special attention; our team has experience collecting data from diverse subjects. To ensure complete and reliable data, we have chosen measures that have been used in nursing homes and are at a sixth grade reading level. Instructions for completing the questionnaires have been written to reflect Oskamp's [78] approaches to reducing response set bias due to social desirability. To ensure confidentiality, participants can place completed surveys directly in a secure drop box in the nursing home. Because surveys will be completed four times, we will change the order of the items each time to reduce the likelihood that respondents will rely on memory of previous responses.

#### Data collection from residents

A list of eligible residents who have fallen in the study periods will be obtained from the minimum data set (MDS) nurse or the falls coordinator. We will select a sample of residents via a random number generator for chart abstraction. We have obtained a waiver of Health Insurance Portability and Accountability Act of 1996 authorization and informed consent for resident chart abstraction for the falls-related process measures.

#### Falls data sources and abstraction timing

Data sources include MDS, resident medical record, medication administration records, fall or incident logs, and administrative facility bed-occupancy rates. All data sources will be examined over the six months preceding study initiation and six months following the FALLS intervention. Medical records are retained in the nursing home by law for at least two years after resident discharge. The timing of abstraction is indicated in Table 3[52-62].

**Table 3:** Fall measures, data sources, calculation, and time points

Concept Measured; Source	Calculation/Definition	Includes six months prior to baseline	Includes six months after FALLS ends
Demographics; Medical record	Gender, Age, and Race. Nominal	X	X

Fall rate; medical record, MDS, incident reports, census	Numerator: number of falls occurring in a 6-month period Denominator: number of occupied facility bed days	X	X

Probability of recurrent falls; as above	Proportion of residents with two or more falls occurring in a 6 month period	X	X

Fall risk reduction; medical record, MDS, incident reports	Count of documented fall risk reduction indicators defined below	X	X

a) Orthostatic Blood Pressure	Documentation of blood pressure in two positions, OR discontinuing medication, adding volume expanding medication, compression stockings	X	X

b) Sensory Impairment	Documentation of presence or absence of visual impairment, OR Intervention to change corrective devices, add assistive technology to optimize sensory input	X	X

c) Footwear	Documentation that footwear was evaluated, modified, or recommended to patient	X	X

d) Gait and Assistive Devices	Physical therapy assessment or training, change in assistive device, or participation in restorative ambulation program	X	X

e) Toileting	Documentation of scheduled toileting or a previous attempt in residents with at least intermittent urinary or bowel continence	X	X

f) Environment	Documentation of a search for environmental factors contributing to fall risk (e.g., low toilet seat, room clutter, burned out light bulb) OR a change in environment likely to reduce falls or injury risk, including repairing grab bars, changing floor surfaces, changing lighting, re-arranging furniture, using a low bed or floor mat, and alarms	X	X

g) Psychotropic Medication Reduction [53-62]	Dose reduction or discontinuation of any of the following classes of psychoactive medications within 1 month of a fall; benzodiazepines, tricyclic antidepressants, antipsychotics, propoxyphene, and selected anticholinergic agents (diphenhydramine, sedating antihistamines, immediate-release oxybutynin, skeletal muscle relaxants)	X	X

h) Calcium and Vitamin D	Prescription of at least 1,000 mg of calcium daily or 800 IU of vitamin D daily, OR equivalent dose regimens. Multivitamins containing vitamin D and combination calcium/vitamin D preparations will be added to the total daily dose calculation.	X	X

#### Abstractor qualifications, training, and blinding

Data abstractors will hold clinical degrees and will be trained using practice charts and a manual including definitions, data locations, and detailed instructions. Instruction will be repeated until inter-rater reliability exceeds 90%. Data collectors are employed by CCME and will be blinded to the nursing home's intervention status and study hypotheses. Blinding will be assessed by asking data collectors to indicate which study group they believe the nursing home was assigned.

#### Data reliability

To ensure data quality, a random 5% of resident charts at each time period will be abstracted by a second data collector, with inter-rater reliability calculated using kappa. Refresher training will be completed if kappa falls below 0.7 for any measure.

#### Measures

The measures and the time points at which these will be collected are summarized in Table 4[79,17,30-93] (Complexity Science Measures) and Table 3 (Fall-related measures). In addition, data will be collected about each nursing home, including bed size, nursing staff hours. Chain and religious affiliation will be collected from publicly available sources http://www.nhcompare.gov. Nursing staff turnover during the intervention period will be obtained from administrators. These data will be used as covariates in the multivariable outcomes analyses. All measures will be aggregated to the cluster level of the facility.

**Table 4:** Complexity science measures

Concept Measured; Source	Psychometrics; Calculation
Demographics; self-report	Age, sex, job title, years in position, education, and ethnicity (collected at baseline or at enrollment into the study. Categorical measurement)

Communication patterns; all staff	Mean scores on Roberts and O'Reilly openness, accuracy scales [79] and Shortell's timeliness scale [80,81]. The scales show adequate reliability and validity in various settings [79,80,82]. In our preliminary studies scales showed reliability alphas of 0.81, 0.72 and 0.68, respectively; construct validity confirmed by factor analysis and hypothesis testing [17,30,83].

NA and LPN participation in decision making about resident care; all staff	Mean score on Anderson et al.'s [83] Participation in Decision-making Instrument (PDMI). The PDMI is established with demonstrated reliability in nursing homes [17,18,83-85] and construct validity established through factor analysis [83] and hypothesis testing [17,84,86]. Nursing home samples achieved alpha coefficients of > 0.90.

Relational Coordination; all staff	Mean scores on Gittell's [87] five-point scale on which staff will rate interactions between groups (e.g., NA to nurse, NA to dietary; nurse to MD). Three aspects are measured including: frequency of communication; high-quality communication; and supportive relationships [88,89]. Gittell [88] adapted this scale for nursing homes and achieved a one factor scale and a Chronbach's alpha 0.86. In our preliminary study, we achieved an alpha of 0.95 on a sample of nursing home staff.

Psychological Safety; All staff	Three items from Edmondson's 7-point psychological safety scale that were modified for heath care [90]. The items ask about whether people are comfortable checking with each other or asking questions, whether people value others' unique skills and talents, and whether people are able to bring up problems and tough issues [91]. Studies in healthcare settings reported alphas of 0.74 [91] and 0.73 [92] We slightly revised the scale by changing the word 'unit' to 'nursing home. Because the scale has not been used in a nursing home sample previously we tested the reading level and found that it read at the 6th grade level, which is acceptable for this low literacy sample.

Safety organizing scale; all staff participants	Mean score on Vogus and Sutcliff's scale designed to measure five 'interrelated behavioral processes: preoccupation with failure, reluctance to simplify interpretations, sensitivity to operations, commitment to resilience, and deference to expertise' [93, p. 47]. In a large sample of hospital RNs, the 9-item, 7-point scale showed reliability (alpha = 0.88), convergent and discriminant validity, and criterion validity, and was reliability aggregated to reflect a unit-level construct [93]. We revised the wording for nursing homes. Alphas were > 0.90 in both the baseline and follow up survey in our preliminary studies.

#### Complexity science measures

Complexity Science Measures (Table 4) will be collected at time points as indicated. We will ask staff to report their experience over the last month; this time frame was chosen to capture the usual monthly cycle of meetings and events that may influence interactions. Although not all staff will have participated in CONNECT, we expect a system effect and, thus, all staff members should perceive changes. Further, we established adequate reliability at the organizational level using ICC, k, Eta-squared, and alpha coefficients on aggregated items scores.

### Fall measures

#### Fall risk factor reduction indicators

Measures chosen for this study are: a component of previous efficacy trials and fall clinical practice guidelines; found to be reliably measured by chart abstraction in previous studies [7,9]; and included in the educational components of the FALLS intervention. These indicators were previously found to be sensitive to change, and not impacted by a ceiling effect [7]. We will calculate the proportion of fallers with medical record evidence of the fall risk reduction indicator, and determine indicator counts for each resident. Timing of the risk factor reduction will be recorded as: within 48 hrs of a fall, within one month of a fall, during the six-month abstraction period. Definitions are found in Table 3.

#### Fall rate

Consistent with the MDS, we define a fall as an unintentional change in position resulting in a resident coming to rest on the ground or lower level [4] regardless of cause [2]. Recurrent falls are defined as two or more falls within the six-month study period [4]. These measures have been successfully employed in previous studies [2-4]. Due to underreporting of falls [94], data will be collected from multiple sources as shown in Table 3. We will calculate fall rates and recurrent falls as defined in the Table 3. From our previous falls study and national data, we assume a baseline fall rate of 1.5 falls/bed/year, and an average bed occupancy rate of 90-bed days/home/month. We therefore project that there will be a total of 2,160 falls in the study nursing homes over the study period. Proportion of repeat fallers and proportion of injurious falls (defined as proportion of falls resulting in injury including skin tear, hematoma, fracture, laceration, need for imaging or urgent assessment) will be measured as secondary fall endpoints.

### Blinding

Because of the nature of the intervention, it is not possible to mask the study assignment from the subjects or the research interventionists. However, the outcomes assessment of falls quality indicators will be completed by independent nurses employed by the state Quality Improvement Organization who will be blinded to study assignment. Success of blinding will be evaluated by asking these nurses to state which intervention they believe that the nursing home received.

### Analysis

The study hypotheses pertain to the cluster level of the nursing homes. Hierarchical linear modeling with Glimmix was used to account for clustering in this study. This procedure is useful when there are multi-level, nested sources of variability such as patients and staff clustering within nursing homes. The Glimmix procedure analyzes both individual and group level trajectories of change over time. Our hypotheses (H) to address the study aims and related analysis are listed below.

#### Aim 1

##### H1A

Residents in facilitates randomized to receive CONNECT + FALLS will have greater improvements in fall risk factor assessment counts from the six-month period preceding the intervention (baseline) to the six-month period after the intervention (follow-up), compared to similar residents in facilities receiving FALLS alone.

##### H1B

Residents in facilities randomized to receive CONNECT + FALLS will have greater improvements in fall risk factor intervention counts from the six-month period preceding the intervention (baseline) to the six-month period after the intervention (follow-up), compared to similar residents in facilities receiving FALLS alone.

As the dependent variables for H1a and H1b are counts, we will use PROC GLIMMIX to estimate the models. A significant negative coefficient will indicate that the intervention reduced fall rates. As is standard practice with Poisson models, we will test for over dispersion in initial analyses and employ a negative-binomial model if over dispersion is present.

#### Aim 2

##### H2A

Residents in facilities randomized to receive CONNECT + FALLS will have lower fall rates, compared to residents in facilities receiving FALLS alone.

##### H2B

Residents in facilities randomized to receive CONNECT + FALLS will have a lower probability of recurrent falls during the six months post-intervention, compared to similar residents in facilities receiving FALLS alone.

##### H2C

Intervention-related improvements in fall rates and injurious falls will be mediated by improvements in fall-related process measures.

The dependent variables for H2a and H2b will consist of one Poisson distributed outcome (fall rates), and one dichotomous outcome (the probability of a recurrent fall). For fall rates, we will use PROC GLIMMIX to re-estimate the model with fall rates dependent and intervention group, time, and relevant covariates as predictors, using the same analysis as for Aim 1. To test H2c, we will add the process measures (from Aim 1) to the models for H2a and H2b as time-changing predictors. We will use bootstrap methods [95] to test significance of these indirect effects.

#### Aim 3

##### H3A

Staff in facilities randomized to receive CONNECT + FALLS will report significantly greater improvement from baseline immediately and three months after the intervention than staff in facilities receiving FALLS alone on complexity science measures of: communication openness, accuracy and timeliness; participation in decision making; relational coordination; psychological safety; and safety culture.

For these outcomes, we will use PROC MIXED to estimate a mixed model to estimates the effect of the intervention on each outcome averaged over time.

##### H3B

Improvements in fall-related process measures and fall-related outcome measures will be mediated by changes in complexity science measures.

To test H3B, we will calculate nursing home-level means on the complexity science measures, add these mean as time-changing predictors to our models (above) predicting fall-related process and outcome measures. We will use bootstrap methods [95] for testing the significance of these indirect effects.

### Statistical power

We used algorithms developed to estimate power for longitudinal models based on the formulae of Jung and Ahn [96], a type I error rate of 0.05 (two-tailed), and a 15% rate of attrition for the staff samples. Hierarchical linear modeling with SAS PROC Glimmix was used to account for clustering in this study. This procedure is useful when there are multi-level, nested sources of variability such as patient and staff clustering within nursing homes. The Glimmix procedure analyzes both individual and group level trajectories of change over time, and can be used to estimate models where persons within clusters are changing over time. Maximum cluster size was limited by the pool of resident fallers and number of staff in each facility. The power analysis algorithms used to determine cluster size take clustering at the individual level into account. In the analyses, we will treat cluster as a fixed rather than as a random effect. This approach will more adequately control on potential confounders at the level of the nursing home. With cluster analyzed as a fixed effect, inter-cluster correlations are not needed to calculate power.

For aim one, we will have 80% power to detect a 15% difference in risk factor assessment and intervention scores, which is considered to be the minimally clinically significant improvement in falls care practice. For aim two, the resident sample will provide 80% power to detect a 23% difference in the fall rate due to intervention, and a 23% difference in the probability of a recurrent fall. Because this is a real world effectiveness study, this change in fall rate is slightly smaller than that seen in a randomized controlled trial of multifactorial risk factor reduction, but still clinically meaningful. For the continuous outcomes in aim three, we will have 80% power to detect standardized differences of 0.21, a magnitude considered small in the statistical literature [97]. As we have a single primary outcome and several additional outcomes that are exploratory, we will not adjust our significance tests for multiple tests.

### Missing data

Item-specific missing data on potential covariates will be handled with maximum-likelihood and multiple imputation techniques [98]. Missing values can be imputed with SAS PROC MI using the Markov chain Monte Carlo algorithm, which can be used with complex missing data patterns as well as for continuous, ordinal, and dichotomous measures [99]. We expect 15% attrition on our dependent variables across waves. Recent work by Chang et al. [100] shows how shared parameter models [101,102] can be used to address potential bias due to a failure to meet the missing at random assumption. These models will be operationalized where necessary.

### Timeline

We were funded (56NR003178) to conduct a pilot study of the CONNECT + FALLS intervention in two nursing homes and FALLS in two nursing homes [31]. We have now been funded (R01NR003178) to conduct this study in 16 additional nursing homes to allow for a full test of the intervention. We will begin nursing home recruitment in fall of 2011 and will stagger recruitment and interventions over four years (completed in 2015). Follow-up data collection and analyses will be completed in 2016.

## Discussion

By focusing on improving local interaction behaviors, we propose that CONNECT is an innovative way to target the learning environment and maximize nursing home staff's ability to adopt content learned in a falls QI program and integrate it into knowledge and action. Preliminary results from the study suggest that local interaction behaviors can be improved in ways that effectively enable the staff to adopt evidence-based current practice for falls prevention [19,31]. We are confident that the capacity exists in most nursing homes to develop and focus these behaviors using existing staff and resources using CONNECT to enhance staff members' abilities to adopt QI interventions in the FALLS program. Because CONNECT is a systems intervention, it can be applied in future projects to examine adoption of other evidence-based practices for a wide variety of clinical problems such as pressure ulcers, pain, and depression and may apply to other healthcare setting.

## Competing interests

The authors declare that they have no competing interests.

## Authors' contributions

RAA and CC-E wrote the protocol in consultation with RMcD. KP and KD reviewed the protocols, executed them and assisted in refining the written protocols from the original proposal. All authors read and approved the final manuscript.

## Authors' information

Ruth A. Anderson, RN, PhD, FAAN, is the Virginia Stone Professor of Nursing at Duke University School of Nursing. Kirsten Corazzini, PhD, Social Gerontologists is Associate Professor at Duke University School of Nursing. Kristie Porter, MPH, is Project Coordinator for the study and Kathryn Daily is a Research Interventionist for this study. Reuben McDaniel is a Professor of Management Science and Information Systems and the Charles and Elizabeth Prothro Chair in Healthcare Management at the College of Business, The University of Texas at Austin, Austin, Texas. Cathleen Colón-Emeric, MD, MHSc, is Associate Professor of Medicine, Department of Geriatrics and is Staff Physician at Croasdaile Village Retirement Community, and Associate Director of the Durham VA Geriatric Research Education and Clinical Center, Durham. Anderson, Corazzini and Colón-Emeric are also Senior Fellows in the Duke University Center for the Study of Aging and Human Development.
